# The Association Between COVID-19-related Discrimination and Probable Post-traumatic Stress Disorder Among Patients With COVID-19 in Sapporo, Japan

**DOI:** 10.2188/jea.JE20230360

**Published:** 2024-12-05

**Authors:** Isaku Kurotori, Toshiaki R. Asakura, Takashi Kimura, Miyuki Hori, Mariko Hosozawa, Masayuki Saijo, Hiroyasu Iso, Akiko Tamakoshi

**Affiliations:** 1 Department of Public Health, Hokkaido University Faculty of Medicine, Sapporo, Japan; 2 Department of Infectious Disease Epidemiology and Dynamics, London School of Hygiene & Tropical Medicine, London, United Kingdom; 3 Centre for Mathematical Modelling of Infectious Diseases, London School of Hygiene & Tropical Medicine, London, United Kingdom; 4 School of Tropical Medicine and Global Health, Nagasaki University, Nagasaki, Japan; 5 Institute for Global Health Policy Research, Bureau of International Health Cooperation, National Center for Global Health and Medicine, Tokyo, Japan; 6 Public Health Office, Health and Welfare Bureau, Sapporo Municipal Government, Sapporo, Japan

**Keywords:** COVID-19, discrimination, PTSD, SARS-CoV-2

## Abstract

**Background:**

Disasters such as earthquakes, terrorism, and pandemics have triggered post-traumatic stress disorder (PTSD), and discrimination against the affected individuals has been linked to the development of PTSD. However, there is limited evidence regarding the association between discrimination against coronavirus disease 2019 (COVID-19) patients and probable PTSD in Japan.

**Methods:**

We conducted a cross-sectional study utilizing a web-based questionnaire targeting individuals who had contracted the severe acute respiratory syndrome-related coronavirus 2 (SARS-CoV-2) infection in Sapporo City. A total of 4,247 individuals with laboratory-confirmed SARS-CoV-2 infection spanning from February 2020 to February 2022 completed the questionnaire (response rate: 15.9%). Probable PTSD was measured using the three-item Posttraumatic Diagnostic Scale. The stratified exact logistic regression was applied to calculate the odds ratios (ORs) of probable PTSD for COVID-19-related discrimination with adjusted factors.

**Results:**

This study included 3,626 patients who had a history of SARS-CoV-2 infection. Among them, 321 patients (8.9%) experienced COVID-19-related discrimination. The prevalence of probable PTSD was 19.6% (63/321) among the patients who experienced COVID-19-related discrimination, and 4.6% (152/3,305) among those who had not encountered such discrimination. The adjusted OR of COVID-19-related discrimination for probable PTSD was 4.68 (95% confidence interval [CI], 3.36–6.53). The population attributable fraction of probable PTSD attributable to COVID-19-related discrimination among COVID-19 patients was estimated to be 23.4% (95% CI, 21.5–25.3%).

**Conclusion:**

The comprehensive epidemiological survey of COVID-19 patients in Japan showed that COVID-19-related discrimination was associated with a higher prevalence of probable PTSD. Mitigating discrimination could be helpful to attenuate PTSD in future pandemics.

## INTRODUCTION

Traumatic events, such as disasters and epidemics, can lead to post-traumatic stress disorder (PTSD).^1^^–^^3^ PTSD presents a public health concern due to its impact on an individual’s social functioning.^4^ A review article indicated that the prevalence of probable PTSD among patients affected with severe acute respiratory syndrome (SARS) in the post-illness stage (a mean follow-up of 33.6 months) was 32.2% (95% confidence interval [CI], 23.7–42.0%).^2^ Globally, until March 2022, more than 481.7 million patients of SARS-related coronavirus 2 (SARS-CoV-2) and over 6.1 million deaths had been reported.^5^ SARS-CoV-2 infection, particularly during the early phases of the pandemic, constituted a life-threatening and traumatic event. A review article found that 16% (95% CI, 9–23%) of patients following severe coronavirus disease 2019 (COVID-19) were at risk of PTSD.^6^ Another review article reported a probable PTSD prevalence of 17.9% (95% CI, 11.6–25.3%) among all survivors who recovered from the acute-phase of COVID-19.^7^ Subgroup analysis revealed a gradual reduction in probable PTSD among COVID-19 patients, although a substantial proportion continued to be affected (23.1%; 95% CI, 10.9–37.9% within <3 months from infection, 16.2%; 95% CI, 9.7–24.0% within 3 to <6 months, and 7.4%; 95% CI, 4.3–11.2% within 6–12 months).^7^ The persistent risk factors for probable PTSD among COVID-19 patients may include hospitalization, quarantine, social isolation, and discrimination.^1^^,^^8^

Discrimination, characterized by unjust behaviors like rejection and exclusion,^9^ can contribute to the development of probable PTSD. A previous study indicated a higher risk of probable PTSD among African Americans with traumatic injuries who experienced racial discrimination in the United States compared with those who did not.^10^ A Japanese study revealed that workers at the Fukushima nuclear power plants after the Great East Japan Earthquake experienced discrimination and had an elevated risk of probable PTSD.^11^ During the early stages of SARS-CoV-2, Asians and Asian Americans encountered COVID-19-related discrimination, including the fear of physical assault, resulting in probable PTSD.^12^

Discrimination was often linked to patients with infectious diseases, such as Hansen’s disease, plague, cholera, yellow fever, SARS, Middle East respiratory syndrome (MERS), Nipah encephalitis, Ebola virus disease, Zika virus disease, and acquired immunodeficiency syndrome (AIDS).^13^^–^^18^ Similarly, discrimination against COVID-19 patients has occurred worldwide without exception.^19^^–^^22^ A mixed-method study revealed that the SARS-CoV-2 outbreak led to region-based and disease-related discrimination against individuals living in a SARS-CoV-2 endemic area and those suspected of having the virus.^23^ Another study showed that COVID-19 patients experienced significantly more COVID-19-related discrimination compared to individuals not infected with the virus from the same region.^24^ This discrimination was associated with persistent psychiatric symptoms after recovery from primary SARS-CoV-2 infection, such as mood symptoms,^25^ sleep disturbance,^26^^,^^27^ anxiety,^25^^,^^28^ psychological distress,^29^^,^^30^ and probable PTSD.^31^^–^^33^ A previous study conducted exclusively in Columbia between October 2020 and April 2021 showed that social discrimination against COVID-19 patients resulted in odds ratios (OR) of approximately 2.5 or higher for probable PTSD.^33^ However, that study adjusted only for age, gender, and income, so other covariates, such as educational attainment,^3^ healthcare work status,^3^ hospitalization,^34^ and previous psychiatric illnesses, should be considered.^35^^,^^36^ A previous study conducted in Japan explored the association between COVID-19-related discrimination and psychological distress (the Kessler Psychological Distress Scale) during the period from April 2020 to November 2021, without a specific focus on probable PTSD.^29^^,^^30^

Therefore, this study aimed to investigate the association between COVID-19-related discrimination and probable PTSD among COVID-19 patients infected between February 2020 and February 2022. Our study considered hospitalization, and history of psychiatric illnesses due to COVID-19 as contributing factors.

## METHODS

### Participants and data collection

We conducted a cross-sectional study involving individuals aged 20–64 years who had been confirmed to be affected with SARS-CoV-2 by reverse-transcriptase polymerase chain reaction (RT-PCR) or COVID-19 antigen test of designated hospitals in Sapporo, the fifth-largest metropolis in Japan with a population of two million. The study participants were extracted from the registry of the Public Health Office of Health & Welfare Bureau of Sapporo Municipal Government up to February 1, 2022, excluding those who had passed away or relocated from Sapporo. On February 21, 2022, or March 3, 2022, we distributed documents to the target individuals containing an individualized web link and a password for a self-report anonymous questionnaire. The study’s purpose was outlined on the first page of the web questionnaire. The study was conducted with voluntary participation, ensuring privacy, maintaining confidentiality, and obtaining digital informed consent from all participants. The deadline for receiving completed questionnaires was set for April 20, 2022. This study was conducted by the ethical principles outlined in the Declaration of Helsinki and received approval from the ethical review board for life science and medical research, Hokkaido University Hospital (no. Sei021-0190). No financial incentives were provided to participants for completing the questionnaire.

### Exclusion criteria of this study

The exclusion criteria were as follows: response after April 1, 2022, more than three years of the age difference between the questionnaire and the registry data, response within a month (30 days) after SARS-CoV-2 infection to differentiate PTSD and acute stress reactions, infection more than once, nonbinary gender, missing values.

### Assessment of probable PTSD

We assessed PTSD symptoms using the three-item Posttraumatic Diagnostic Scale (PDS), a validated scale derived from PDS.^37^^–^^39^ PDS aligns with the PTSD criteria, which are outlined in the Diagnostic and Statistical Manual of Mental Disorders, fourth edition.^40^ The three-item PDS evaluated the DSM-IV criteria B1 (intrusive images and thoughts), B2 (nightmares), and B5 (physiological reactions to trauma reminders) related to COVID-19, focusing on the symptoms in the past month preceding questionnaire completion. Each item employed a 4-point scale (0 = “not at all or only one time” to 3 = “five or more times a week/almost always”), with the total score ranging from 0 to 9 points. We categorized participants with a total score of 3 or more as probable PTSD.^39^ The three-item PDS demonstrated a sensitivity of 94.8% and a specificity of 86.1% in individuals with PTSD, as determined by the Clinician-Administered PTSD scale interview.^39^

### COVID-19-related discrimination

We evaluated COVID-19-related discrimination with the following question we devised: “Have you ever perceived bullying, discrimination, or unfair treatment because of your SARS-CoV-2 infection?” Participants could respond with a “yes” or “no”. If participants answered “yes,” they were subsequently asked, “From whom or in what situation have you encountered discrimination?” The available response options included family, relatives, friends, neighborhood, workplace, and school.

### Questionnaire on the sociodemographic characteristics and clinical information

We collected following variables: gender, age group (stratified by decade), educational attainment (high school or less, technical school, junior/professional training college, or university or above), annual household income (less than two million Japanese yen [JY], two to four million JY, four to eight million JY, eight million or more JY, or preferred not to answer; as of February 2022, 100 Japanese yen was approximately equivalent to 0.87 the United States dollar), marital status (unmarried/divorced/widowed, or married/in a de facto relationship), prior status as a healthcare worker (yes or no), and history of psychiatric illnesses (PTSD, depression, bipolar disorder, anxiety disorder, dementia, or others) before the onset of COVID-19. Information concerning the duration of SARS-CoV-2 infection, the date of SARS-CoV-2 positive confirmation, and hospitalization status (whether hospitalized or not upon infection) was retrieved from the registry data of the Sapporo City Public Health Office. Clinical data from the registry was anonymously integrated into the questionnaire data using unique linkage keys. The Sapporo Municipal Government defined the pandemic waves of SARS-CoV-2 infection as follows: Wave 1 (February 14 to April 7, 2020), Wave 2 (April 8 to October 27, 2020), Wave 3 (October 28, 2020, to March 7, 2021), Wave 4 (March 8 to July 11, 2021), Wave 5 (July 12, 2021, to January 6, 2022), and Wave 6 (January 7 to June 26, 2022). The deadline for response in this study was April 20, 2022, which was in the middle of Wave 6. In our analysis, we combined Waves 1, 2, and 3 due to the small number of patients in each wave.

### Statistical analyses

We investigated the association between COVID-19-related discrimination and probable PTSD among COVID-19 patients. Each categorical variable underwent testing using either the chi-square test or Fisher’s exact test. Stratified exact logistic regression, with the pandemic waves of SARS-CoV-2 infection as a strata variable, was utilized to calculate the crude OR, two adjusted ORs, and 95% CI. In model 1, adjustments were made for gender, age group, and history of psychiatric illnesses. In model 2, additional adjustments included hospitalization, educational attainment, annual household income, marital status, and prior status as a healthcare worker. The logistic regression was conducted to estimate the OR within each pandemic wave. We also conducted multivariable analysis of variance to show least square means and associations between COVID-19-related discrimination and a three-item PDS total score as a sensitivity analysis. Furthermore, we estimated the population attributable fraction (PAF) and 95% CI, adjusted for gender and age group among the COVID-19 patients. PAF was estimated by PAF = P_e_(OR − 1)/OR. Here, P_e_ represents the proportion of exposure among the patients.

All statistical comparisons were two-tailed, and *P*-values <0.05 were considered to be statistically significant. All data assessments were performed using SAS software (version 9.4; SAS Institute Inc., Cary, NC, USA).

## RESULTS

### Sociodemographic characteristics of the COVID-19 patients

**Figure 1.**
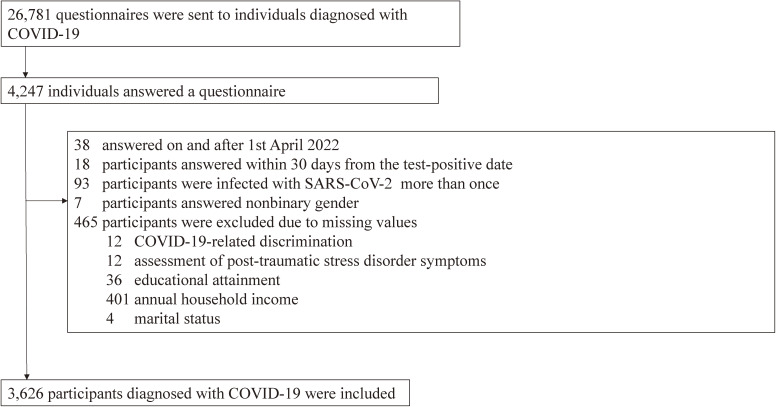
Flowchart of inclusion and exclusion criteria of COVID-19 patients in the present study. COVID-19, coronavirus disease 2019.

The sociodemographic characteristics are presented in Table 1. A total of 321 patients (8.9%) encountered COVID-19-related discrimination. We received questionnaires from 4,247 laboratory-confirmed COVID-19 patients (response rate: 15.9% [4,247/26,781]). We excluded patients according to the exclusion criteria, as shown Figure 1. More than 3 years of the age difference between the questionnaire and the registry data was not found. For our analysis, 3,626 patients were included. The last participant patient in this study had a SARS-CoV-2 positive confirmation date of February 8, 2022.

**Table 1.** Characteristics of COVID-19 patients according to the experience of COVID-19-related discrimination

	COVID-19-related discrimination				*P*-value^a^
	Yes		No		
	*N* = 321		*N* = 3,305		
Gender					0.062
Female	198	(61.7%)	1,860	(56.3%)	
Male	123	(38.3%)	1,445	(43.7%)	
Age group, years					0.070
20–29	53	(16.5%)	573	(17.3%)	
30–39	66	(20.6%)	715	(21.6%)	
40–49	105	(32.7%)	861	(26.1%)	
50–59	97	(30.2%)	1,156	(35.0%)	
Hospitalization due to infection					0.224
Hospitalized	80	(24.9%)	726	(22.0%)	
Not hospitalized	241	(75.1%)	2,579	(78.0%)	
Wave including SARS-CoV-2 positive confirmation date^b^					0.003
Waves 1, 2, and 3	115	(35.8%)	866	(26.2%)	
Wave 4	95	(29.6%)	1,116	(33.8%)	
Wave 5	85	(26.5%)	966	(29.2%)	
Wave 6	26	(8.1%)	357	(10.8%)	
Educational attainment					0.012
High school or lower	126	(39.3%)	1,021	(30.9%)	
Technical school	67	(20.9%)	702	(21.2%)	
Junior/Professional training college	40	(12.5%)	442	(13.4%)	
University or higher	88	(27.4%)	1,140	(34.5%)	
Annual household income (per million Japanese yen)					0.039
<2	68	(21.2%)	510	(15.4%)	
≥2 to <4	79	(24.6%)	855	(25.9%)	
≥4 to <8	118	(36.8%)	1,235	(37.4%)	
≥8	56	(17.5%)	705	(21.3%)	
Marital status					0.021
Unmarried/Divorced/Widowed	150	(46.7%)	1,326	(40.1%)	
Married/In a de facto relationship	171	(53.3%)	1,979	(59.9%)	
Prior status as a healthcare worker					0.337
Yes	43	(13.4%)	383	(11.6%)	
No	278	(86.6%)	2,922	(88.4%)	
History of psychiatric illnesses^c^					0.012
Yes	31	(9.7%)	200	(6.1%)	
No	290	(90.3%)	3,105	(94.0%)	

COVID-19, coronavirus disease 2019; SARS-CoV-2, severe acute respiratory syndrome-related coronavirus 2.

^a^Chi-square test.

^b^Waves 1, 2, and 3: February 14, 2020, to March 7, 2021; Wave 4: March 8 to July 11, 2021; Wave 5: July 12, 2021, to January 6, 2022; Wave 6: January 7 to June 26, 2022. The last participant patient’s data was recorded on February 8, 2022.

^c^Posttraumatic stress disorder, depression, bipolar disorder, anxiety disorder, dementia, and any other psychiatric illnesses.

The patient count of SARS-CoV-2 positive confirmations in each pandemic wave was as follows: 981 patients (Waves 1, 2, and 3), 1,211 patients (Wave 4), 1,051 patients (Wave 5), and 383 patients (Wave 6). The count (percentage) of COVID-19-related discrimination in each pandemic wave was as follows: 115 patients (11.7%) in Waves 1, 2, and 3, 95 patients (7.8%) in Wave 4, 85 patients (8.1%) in Wave 5, and 26 patients (6.8%) in Wave 6. The median duration after SARS-CoV-2 infection among patients who encountered COVID-19-related discrimination and those who did not was 318 (standard deviation [SD], 153) days and 286 (SD, 138) days. Median duration after SARS-CoV-2 infection among patients who encountered COVID-19-related discrimination and those who did not in each pandemic wave was as follows: 488 (SD, 85) days and 468 (SD, 72) days (Waves 1, 2, and 3), 295 (SD, 28) days and 298 (SD, 24) days (Wave 4), 198 (SD, 33) days and 197 (SD, 29) days (Wave 5), and 47 (SD, 9) days and 45 (SD, 9) days (Wave 6), respectively.

The proportion of females with COVID-19-related discrimination and those not was 61.7% (198/321) and 56.3% (1,860/3,305). The median age of the COVID-19 patients who encountered COVID-19-related discrimination and those who did not was 42.9 (SD, 11.2) years and 43.2 (S, 11.9) years. Patients who encountered COVID-19-related discrimination were significantly related to an earlier pandemic wave of SARS-CoV-2 infection, lower educational attainment, lower annual household income, unmarried/divorced/widowed status, and a higher prevalence of history of psychiatric illnesses compared with those who did not.

### Situations in which the COVID-19 patients encountered discrimination

The proportion of each situation in which the COVID-19 patients encountered discrimination was the following (Table 2): 80.7% (*n* = 259) in workplaces, 11.5% (*n* = 37) from friends, 10.6% (*n* = 34) from family members, 6.2% (*n* = 20) from relatives, 5.6% (*n* = 18) in the neighborhood, and 3.4% (*n* = 11) in schools.

**Table 2.** Situations in which COVID-19 patients encountered discrimination

Situation	Distribution of COVID-19-related discrimination		Prevalence of COVID-19-related discrimination according to the status of probable post-traumatic stress disorder				*P*-value^a^
			Yes		No		
Total	*N* = 321		*N* = 215		*N* = 3,411		
Workplace	259	(80.7%)	53	(24.7%)	206	(6.0%)	<0.001
Friend	37	(11.5%)	7	(3.3%)	30	(0.9%)	<0.001
Family	34	(10.6%)	8	(3.7%)	26	(0.8%)	<0.001
Relative	20	(6.2%)	5	(2.3%)	15	(0.4%)	<0.001
Neighborhood	18	(5.6%)	3	(1.4%)	15	(0.4%)	0.053
School	11	(3.4%)	3	(1.4%)	8	(0.2%)	0.003

COVID-19, coronavirus disease 2019.

^a^Fisher exact test.

### Association between COVID-19-related discrimination and probable PTSD

The prevalence of probable PTSD was 5.9% (215/3,626) as a whole, 19.6% (63/321) among the COVID-19 patients who encountered COVID-19-related discrimination, and 4.6% (152/3,305) among those who did not. The corresponding prevalence in each pandemic wave was as follows: 14.8% (17/115) and 5.0% (43/866) in Waves 1, 2, and 3, 24.2% (23/95) and 5.5% (61/1,116) in Wave 4, 17.6% (15/85) and 4.1% (40/966) in Wave 5, and 30.8% (8/26) and 2.2% (8/357) in Wave 6. The stratified exact logistic regression analysis with the pandemic waves of SARS-CoV-2 infection revealed that COVID-19-related discrimination was significantly associated with probable PTSD (OR 5.08; 95% CI, 3.69–7.01 in the crude model, OR 4.90; 95% CI, 3.55–6.78 in model 1, and OR 4.68; 95% CI, 3.36–6.53 in model 2) (Table 3). The corresponding OR in each pandemic wave was as follows: 2.98 (95% CI, 1.59–5.59) in Waves 1, 2, and 3, 5.65 (95% CI, 3.20–9.99) in Wave 4, 4.74 (95% CI, 2.40–9.35) in Wave 5, and 29.9 (95% CI, 6.40–139.31) in Wave 6 after model 2 adjustment. The multivariable analysis of variance also showed a significant association between COVID-19-related discrimination and a three-item PDS total score across all waves and within each wave (eTable 1). The estimated PAF, probable PTSD attributable to COVID-19-related discrimination, was 23.4% (95% CI, 21.5–25.3%) adjusted for gender and age group. The corresponding PAF in each pandemic wave was 19.6% (95% CI, 14.3–24.9%) in Waves 1, 2, and 3, 22.5% (95% CI, 19.8–25.1%) in Wave 4, 21.7% (95% CI, 18.0–25.3%) in Wave 5, and 47.6% (95% CI, 44.7–50.5%) in Wave 6.

**Table 3.** Odds ratios and population attributable fraction associating COVID-19-related discrimination with probable post-traumatic stress disorder

Wave including SARS-CoV-2 positive confirmation date^a^	COVID- 19-related discrimination	Probable post-traumatic stress disorder/ number at risk	Crude model	Model 1^b^	Model 2^b^	Population attributable fraction^c^ (95% CI)
			OR (95% CI)	OR (95% CI)	OR (95% CI)	
Overall period	No	152/3,305	ref.	ref.	ref.	
	Yes	63/321	5.08 (3.69–7.01)^*^	4.90 (3.55–6.78)^*^	4.68 (3.36–6.53)^*^	23.4% (21.5–25.3%)^*^
Waves 1, 2, and 3	No	43/866	ref.	ref.	ref.	
	Yes	17/115	3.32 (1.82–6.05)^*^	3.25 (1.77–5.96)^*^	2.98 (1.59–5.59)^*^	19.6% (14.3–24.9%)^*^
Wave 4	No	61/1,116	ref.	ref.	ref.	
	Yes	23/95	5.53 (3.23–9.44)^*^	5.54 (3.22–9.52)^*^	5.65 (3.20–9.99)^*^	22.5% (19.8–25.1%)^*^
Wave 5	No	40/966	ref.	ref.	ref.	
	Yes	15/85	4.96 (2.61–9.42)^*^	4.84 (2.54–9.25)^*^	4.74 (2.40–9.35)^*^	21.7% (18.0–25.3%)^*^
Wave 6	No	8/357	ref.	ref.	ref.	
	Yes	8/26	19.39 (6.53–57.58)^*^	18.35 (5.37–62.66)^*^	29.9 (6.40–139.31)^*^	47.6% (44.7–50.5%)^*^

CI, confidence interval; COVID-19, coronavirus disease 2019; OR, odds ratio; SARS-CoV-2, severe acute respiratory syndrome-related coronavirus 2.

^a^Waves 1, 2, and 3: February 14, 2020 to March 7, 2021; Wave 4: March 8 to July 11, 2021; Wave 5: July 12, 2021 to January 6, 2022; Wave 6: January 7 to June 26, 2022. The last participant patient’s data was recorded on February 8, 2022.

^b^Model 1: adjusted for gender, age group, and history of psychiatric illnesses; model 2: adjusted for all variables in model 1, hospitalization, educational attainment, annual household income, marital status, and prior status as a healthcare worker due to COVID-19.

^c^We estimated the population attributable fraction with adjustment for gender and age group. The stratified exact logistic regression with the pandemic waves of SARS-CoV-2 infection as a strata variable was applied among all. The logistic regression was conducted to estimate the OR within each pandemic wave.

^*^P < 0.001.

## DISCUSSION

This study represents a comprehensive epidemiological survey aimed at identifying the association between COVID-19-related discrimination and probable PTSD in COVID-19 patients following SARS-CoV-2 infection after the year 2021 in Japan. There were approximately 5 times higher OR of probable PTSD in COVID-19 patients who encountered COVID-19-related discrimination compared with those who did not through multivariable adjustment for potential covariants including social status and history of psychiatric illnesses. The association remained significant across all models, and the OR decreased only slightly following the adjustment of covariates. Furthermore, our findings suggest that if COVID-19-related discrimination were eliminated, 23.4% (95% CI, 21.5–25.3%) of patients with probable PTSD would be preventable among COVID-19 patients.

Our study revealed that patients infected at Waves 1, 2, and 3 of the pandemic encountered more than 10% of COVID-19-related discrimination, with a gradual decrease in prevalence in the subsequent waves. In the initial phases of the outbreak, COVID-19-related discrimination stemmed from fear of infection propagated through mass media and social platforms.^41^ A prior Japanese study conducted during an earlier survey period than ours found that 32.7% of COVID-19 patients experienced stigma and discrimination.^29^ In March 2020, the Japanese government prioritized efforts to combat unjust social discrimination against COVID-19 patients and healthcare workers.^42^ Simultaneously, organizations such as the United Nations International Children’s Emergency Fund, the World Health Organization, and the International Federation of Red Cross and Red Crescent Societies issued declarations aimed at preventing social discrimination in the early stages of the outbreak.^43^ Recognizing the imperative to prevent social discrimination is crucial for public health measures in both current and future pandemics.^44^^,^^45^

We also showed that COVID-19-related discrimination was associated with probable PTSD after the year 2022. In this study, the high prevalence of probable PTSD maintained among the COVID-19 patients who encountered COVID-19-related discrimination compared with those who did not, in contrast to a previous study that showed a gradual decline (23.1%; 95% CI, 10.9–37.9% within <3 months from infection, 7.4%; 95% CI, 4.3–11.2% within 6–12 months).^7^ A 2-year cohort study in China showed that COVID-related trauma and low social support were risk factors for probable PTSD in COVID-19 patients,^46^ and our result may be linked to these two risks. Moreover, a recent study showed that COVID-19-related discrimination on psychological distress was more severe among those infected in later phases.^30^ While the interpretation of the adjusted OR of probable PTSD in Wave 6 requires careful consideration, our findings emphasize the importance of preventing social discrimination, not only in the initial phases of the pandemic but throughout the entire pandemic duration.

According to our study, approximately 80% of COVID-19-related discrimination predominantly occurred in the workplace. Previous studies of veterans or peacekeepers exposed to wartime experiences highlighted the association between negative homecoming experiences and probable PTSD.^47^^,^^48^ Negative perceptions within the community regarding those who had experienced wartime trauma may dissuade them from openly sharing their thoughts and emotions regarding traumatic wartime events, potentially intensifying probable PTSD.^49^ A previous study suggested that discrimination against affected individuals was linked to social isolation, worsening symptoms of PTSD, and could also lead to feelings of guilt about contracting the virus, which might discourage them from seeking treatment and could worsen their symptoms of PTSD.^4^ Our findings underscore the pressing need to persist in efforts aimed at diminishing discrimination specifically in workplaces.

The present study has several limitations. First, our study was cross-sectional, and we carefully made conclusions about the causal relationship. However, it is unlikely that probable PTSD causes COVID-19-related discrimination. Second, the response rate of participants was low, and selection biases were potentially included in this study. COVID-19 patients with more serious symptoms of PTSD might be resistant to answering questionnaires to avoid reminders of traumatic events, leading to potential underestimation. Third, we used self-reporting questionnaires, which may introduce recall biases and lead to exaggerated reports of COVID-19-related discrimination experiences. Additionally, a questionnaire we used to assess COVID-19-related discrimination was original and not validated. Fourth, the definition of probable PTSD in this study was not based on clinical evaluation or confirmed diagnoses by trained psychiatrists, although the three-item PDS was used in another article as a validated scale.^50^ Fifth, we did not ask whether healthcare workers were engaged in frontline work related to COVID-19-related discrimination. However, a previous study showed an association between COVID-19-related discrimination and a three-item PDS total score remained unaltered even after adjustment with frontline work status.^50^ Finally, we did not have information on personal factors, such as childhood adversity, resilience to traumatic events, genetic variants of SARS-CoV-2, differences in disease severity and treatment during acute-phase or post-acute infection, length of hospital stay, and SARS-CoV-2 vaccination history.

In conclusion, we found a higher prevalence of probable PTSD in COVID-19 patients who encountered COVID-19-related discrimination compared with those who did not. Although further studies are necessary to confirm the impact of COVID-19-related discrimination on PTSD among COVID-19 patients, mitigating the discrimination could be helpful to attenuate PTSD in future pandemics.

## SUPPLEMENTARY MATERIAL

eTable 1. Association between COVID-19-related discrimination and a three-item Posttraumatic Diagnostic Scale total score
