# Supplementary material for: The Association Between COVID-19-related Discrimination and Probable Post-traumatic Stress Disorder Among Patients With COVID-19 in Sapporo, Japan
Source: J Epidemiol. 2024 Dec 5;34(12):570–6. doi: 10.2188/jea.JE20230360 (PMC11564067; doi:10.2188/jea.JE20230360)
Supplement: Supplementary file 1 [file je-34-570-s001.pdf]

eTable 1. Association between COVID-19-related discrimination and a three-item Posttraumatic Diagnostic Scale total score

Wave including SARS-CoV-2 positive confirmation date[a]	COVID-19-related discrimination	Crude model	Model 1[b]	Model 2[b]
		LSM (95% CI)	LSM (95% CI)	LSM (95% CI)
Overall period	No	0.39 (0.35–0.42)	0.47 (0.40–0.54)	0.60 (0.51–0.69)
	Yes	1.18 (1.07–1.29)*	1.25 (1.12–1.37)*	1.35 (1.21–1.48)*
Waves 1, 2, and 3	No	0.44 (0.36–0.51)	0.43 (0.28–0.58)	0.56 (0.39–0.74)
	Yes	1.01 (0.80–1.22)*	0.99 (0.75–1.23)*	1.09 (0.83–1.34)*
Wave 4	No	0.40 (0.34–0.46)	0.47 (0.35–0.59)	0.57 (0.42–0.73)
	Yes	1.38 (1.17–1.59)*	1.44 (1.21–1.67)*	1.52 (1.27–1.77)*
Wave 5	No	0.36 (0.30–0.42)	0.53 (0.41–0.66)	0.70 (0.53–0.87)
	Yes	1.04 (0.83–1.24)*	1.20 (0.97–1.43)*	1.32 (1.06–1.58)*
Wave 6	No	0.29 (0.21–0.37)	0.47 (0.25–0.69)	0.44 (0.04–0.84)
	Yes	1.65 (1.34–1.96)*	1.76 (1.41–2.11)*	1.76 (1.30–2.22)*

CI, confidence interval; COVID-19, coronavirus disease 2019; LSM, least square means; SARS-CoV-2, severe acute respiratory syndrome-related coronavirus 2.

[a] Waves 1, 2, and 3: February 14, 2020 to March 7, 2021; Wave 4: March 8 to July 11, 2021; Wave 5: July 12, 2021 to January 6, 2022; Wave 6: January 7 to June 26, 2022. The last participant patient's data was recorded on February 8, 2022.

[b] Model 1: adjusted for gender, age group, and history of psychiatric illnesses; model 2: adjusted for all variables in model 1, hospitalization, educational attainment, annual household income, marital status, and prior status as a healthcare worker due to COVID-19

* $P<0.001$
